# Exploring the role of DeepSeek-R1, ChatGPT-4, and Google Gemini in medical education: How valid and reliable are they?

**DOI:** 10.12669/pjms.41.7.12183

**Published:** 2025-07

**Authors:** Sultan Ayoub Meo, Farah A Abukhalaf, Riham A ElToukhy, Kamran Sattar

**Affiliations:** 1 Sultan Ayoub Meo Department of Physiology, College of Medicine, King Saud University, Riyadh, Saudi Arabia; 2 Farah A Abukhalaf College of Medicine, College of Medicine, King Saud University, Riyadh, Saudi Arabia; 3 Riham A. ElToukhy Department of Family and Community Medicine, College of Medicine, King Saud University, Riyadh, Saudi Arabia; 4 Kamran Sattar Department of Medical Education, College of Medicine, King Saud University, Riyadh, Saudi Arabia

**Keywords:** Assessment, ChatGPT, DeepSeek, Google Gemini, Knowledge, Medical Education

## Abstract

**Objective:**

In recent years, Artificial Intelligence (AI) has led to rapid advancements in science, technology, industries, healthcare settings, and medical education. A Chinese-built large language model, DeepSeek-R1, inspires the scientific community as an affordable and open alternative to earlier established US-based AI models, ChatGPT-4 and Google Gemini 1.5 Pro. This study aimed to explore the role of “DeepSeek-R1, ChatGPT-4 and Google Gemini 1.5 Pro” and to assess the validity and reliability of these AI tools in medical education.

**Methods:**

The current cross-sectional study was performed in the Department of Physiology, College of Medicine, King Saud University, Riyadh, Saudi Arabia during the period January 25, 2025, to February 28, 2025. The Multiple-Choice Questions (MCQs) bank was created with a pool of basic medical sciences (60 MCQs) and clinical medical sciences (40 MCQs). The one hundred MCQs were prepared from various medical textbooks, journals, and examination pools. The MCQs were individually entered into the given area of the “DeepSeek-R1, ChatGPT-4 and Google Gemini 1.5 Pro” to assess the level of knowledge in various disciplines of medical sciences.

**Results:**

The marks obtained in basic medical sciences by DeepSeek R1 47/60 (78.33%), ChatGPT-4 47/60 (78.33%), and Google Gemini 1.5 Pro 49/60 (81.7%). However, in clinical medical sciences, the marks obtained by DeepSeek R1 were 35/40 (87.5%), ChatGPT-4 36/40 (90.0%), and Google Gemini 1.5 Pro 33/40 (82.5%). The total marks obtained by DeepSeekR1 were 82/100 (82.0%), Chat GPT-4 84/100 (84.0%), and Google Gemini-1.5 Pro 82/100 (82.0%).

**Conclusions:**

The Chinese-based DeepSeek-R1, the US-based ChatGPT-4, and Google Gemini-1.5 Pro achieved similar scores, exceeding 80% marks, in various medical sciences subjects. The study findings demonstrate that the knowledge, validity, and reliability levels of DeepSeek R1, ChatGPT-4, and Google Gemini 1.5 Pro are similar for their potential future use in medical education.

## INTRODUCTION

In recent years, 2023 and 2024, the world has witnessed that global human society entering the age of artificial intelligence (AI). AI has rapidly reformed science, technology, industries, finance, healthcare settings, and medical education. AI performs complex tasks, analyses vast datasets, and provides predictive insights on complex matters.^1^,^2^

AI has transformed various aspects of human life, from industry automation to specific digital assistants. The most advanced AI models include ChatGPT, Google Gemini, and, more recently, DeepSeek, leading AI chatbots with unique and highly advanced features. A Chinese-made, large language model, DeepSeek-R1, inspires researchers as an affordable and alternative tool to reasoning models, Chat GPT-4.^3^ The Chinese technology has gained significant attention worldwide with the release of DeepSeek-R1, which challenges the performance of the dominant tools developed by the US.^4^

The scientists highlight the importance of DeepSeek, an inexpensive and powerful AI model that amazed the global science community and public after a Chinese firm released it in the third week of January 2025. DeepSeek-R1 can resolve the science and research allied problems that match OpenAI, whose reasoning models are considered by many global influencers. Based on its performance and low cost, it is believed that DeepSeek-R1 encourages researchers and scientists to use it in research without worrying about the cost.^5^

DeepSeek, ChatGPT, and Google Gemini represent innovative AI language models that take distinctly different approaches to solving similar problems. These AI models are the subject of intense discussion in the global scientific community, with a particular focus on health sciences and medical education. Medical education requires highly standardized assessment tools to provide updated knowledge and skills for improved clinical practices.^6^

Academic institutions and accrediting examination bodies frequently use multiple-choice questions (MCQs) to assess knowledge in various scientific disciplines worldwide. MCQs are commonly used in medical examinations as a reliable instrument to encourage the learning and assessment processes.^7^ MCQs are pivotal in evaluating cognitive capacities, critical thinking, and problem-solving abilities.^8^,^9^ MCQs measure the applicant’s ability to connect concepts and analyze evidence in multiple contexts. Moreover, encouraging critical thinking and knowledge application provides a strong framework for assessing higher cognitive functions.^8^,^9^

The science community has mixed opinions about AI knowledge and its reliability and validity. The scientists believe that AI could help summarize research, but it also poses risks.^10^ Many scientists are impressed with their ability, and some doubt that these tools may provide inaccurate information. These tools can produce incorrect citations, misrepresent facts, and fail to distinguish between information.^11^ Therefore, considering all these facts, this study aimed to investigate the levels of knowledge of DeepSeek R1, ChatGPT-4, and Google Gemini 1.5 Pro in medical sciences examinations and assess their validity and reliability.

## METHOD

The current “cross-sectional study was performed in the Department of Physiology, College of Medicine, King Saud University, Riyadh, Saudi Arabia” during the period January 25, 2025, to February 28, 2025.

### Selection of MCQs Examination:

The two research team members arranged the content-wise MCQs from various textbooks, medical journals, web sources, and examination pools. Another team member reviewed the questions and confirmed that their answers were appropriate. Each question was scenario-based and was established on various physiological, anatomical, biological, and pathophysiological aspects, along with analytic characteristics (Table-I). It was also assured that all the questions were well-constructed and clear, without any indications or answer solution suggestions. On five MCQs, a question-based test was piloted on ChatGPT-4, DeepSeek R1, and Google Gemini 1.5 Pro to check for technical issues in the questions. Once the research team was satisfied with the MCQs and their quality, all questions were compiled for the examination. A total of one hundred MCQs in both basic and clinical medical sciences, including Anatomy, Physiology, Biochemistry, Pharmacology, Pathology, Medicine, Obstetrics and Gynaecology and Surgery, were selected from the MCQs pool. The knowledge levels of DeepSeek R1, ChatGPT-4, and Google Gemini 1.5 Pro were assessed.

**Table-I:** Distribution of MCQs in basic medical sciences and clinical medical sciences.

Multiple Choice Questions (MCQs)	Numbers of MCQs
***Basic Medical Sciences***	
Anatomy	10
Embryology	10
Physiology	10
Biochemistry	10
Pharmacology	10
Pathology	10
Subtotal	60
***Clinical Medical Sciences***	
Medicine	10
Surgery	10
Obstetrics and Gynaecology	10
Pediatrics’	10
Subtotal	40
Total	100

### Data Collection:

Each MCQ was individually entered into the designated area in DeepSeek R1, ChatGPT-4, and Google’s Gemini 1.5 Pro, and a new session was initiated for each entry to minimize memory retention bias. The responses and answers were recorded. The initial response was used as the final response, and we did not use the option “regenerate response. Based on the established answer key, the score was calculated on a scale of 0 or 1, where 0 indicated an incorrect response and 1 demonstrated a correct response.

### Inclusion and exclusion criteria:

The scenario-based multiple-choice questions (MCQs) in basic and clinical medical sciences, sourced from various textbooks, medical journals, web sources, and examination pools, were included. All MCQs were established based on physiological, anatomical, biological, and pathophysiological, as well as medical and surgical aspects. MCQs with any indication of the answer, as well as true-and-false-based responses, were excluded from the study. The MCQs piloted on ChatGPT-4, DeepSeek R1, and Google Gemini 1.5 Pro, which were used to check for technical issues in the questions, were also excluded from the study.

### Ethical Approval and Statistical Analysis:

MCQs were compiled using textbooks, academic journals, institutional examination bodies, and databases. This study did not involve animal or human subjects, so ethical permission was not required, even though we obtained an exemption from the Institutional Review Board (IRB) Unit, College of Medicine, KSU (IRB exemption #: E 259577, dated: March 4, 2025).

The data analysis was conducted using SPSS version 30. Data normality was assessed using the Shapiro-Wilk test, which revealed that the score distributions were not normally distributed (p < 0.05 for all models). Since the normality assumption was not met, a non-parametric Kruskal-Walli’s test was selected instead of ANOVA to compare the performance of the three AI models. The Kruskal-Walli’s test was performed individually for each subject to determine whether there were statistically significant differences in model performance. A p-value ≤ 0.05 was considered statistically significant. Where all three models received identical scores, statistical testing was not performed due to the lack of variation. In such cases, a p-value of one was assigned, indicating no difference in performance.

## RESULTS

**Fig.1:**
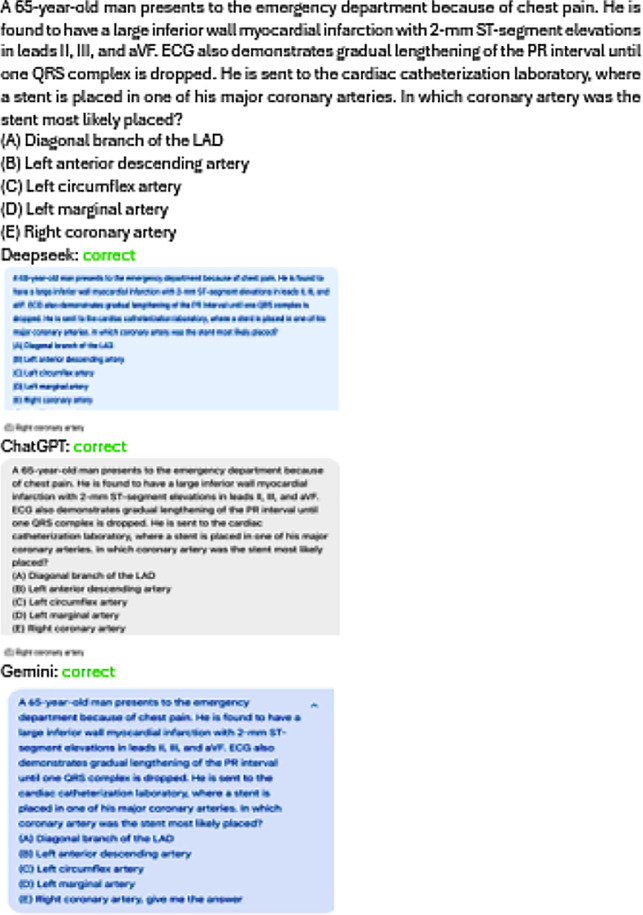
The pattern of MCQs and the response of DeepSeek, ChatGPT and Google Gemini.

The medical knowledge of DeepSeek R1, ChatGPT-4 and Google Gemini 1.5 Pro was assessed based on the ability to respond to individual Multiple-Choice Questions (MCQs) in basic and clinical medical sciences. The MCQs covered distinct areas of medical science based on the medical case scenario. The number of MCQ questions from basic medical sciences was 60 (Table-I). The basic sciences subjects include “Anatomy (10), Embryology (10), Physiology (10), Biochemistry (10), Pharmacology (10), and Pathology (10)”. Similarly, in clinical medical sciences, 40 MCQs were selected; the subject areas included “Medicine (10), Surgery (10), Obstetrics and Gynecology (10), and Pediatrics (10)”. The total number of MCQs in basic and clinical medical sciences was 100 (Table-I). The MCQs were based on medical case-based scenarios (Fig.1).

Table-II demonstrated that out of one hundred MCQs in various health sciences disciplines, DeepSeek R1, ChatGPT-4, and Google Gemini 1.5 Pro attempted all 100 questions. The marks obtained in basic medical sciences by DeepSeek-R1 47/60 (78.33%), ChatGPT-4 47/60 (78.33%), and Google Gemini 1.5 Pro 49/60 (81.7%). However, in clinical medical sciences, the marks obtained by DeepSeek-R1 35/40 (87.5%), ChatGPT-4 36/40 (90.0%), and Google Gemini 1.5 Pro 33/40 (82.5%) (Table-II). The overall total marks obtained by DeepSeek-R1 82/100 (82.0%), ChatGPT-4 84/100 (84.0%), and Google Gemini 1.5 Pro 82/100 (82.0%) (Table-II). All three AI tools — DeepSeek R1, ChatGPT-4, and Google Gemini 1.5 Pro — obtained scores of over 80% in various medical sciences disciplines. However, no significant differences were found between the marks obtained by DeepSeek R1, ChatGPT -4, and Google Gemini 1.5 Pro (Table-II).

**Table-II:** Marks obtained by DeepSeek R1, ChatGPT-4 and Google Gemini 1.5 Pro in various subjects in basic and clinical medical sciences.

Medical Science Subjects	DeepSeek-R1	ChatGPT-4	Google Gemini	Significance level
***Basic Medical Sciences (Marks 60)***				
Anatomy	7/10 (70%)	7/10(70%)	7/10(70%)	p=1.000*
Embryology	7/10 (70%)	9/10 (90%)	8/10(80%)	p=0.368
Physiology	8/10 (80%)	9/10 (90%)	7/10 (70%)	p=0.368
Biochemistry	8/10 (80%)	8/10 (80%)	9/10(90%)	p=0.368
Pharmacology	8/10 (80%)	7/10 (70%)	9/10(90%)	p=0.368
Pathology	9/10 (90%)	7/10 (70%)	9/10(90%)	p=0.368
Marks Obtained	47/60 (78.33)	47/60 (78.33%)	49/60 (81.7%)	p=0.368
***Clinical Medical Sciences (Marks 40)***				
Medicine	8/10(80%)	8/10(80%)	6/10(60%)	p=0.368
Surgery	10/10(100%)	9/10(90%)	9/10(90%)	p=0.368
Obstetrics and gynaecology	8/10(80%)	9/10(90%)	9/10(90%)	p=0.368
Paediatrics	9/10 (90%)	10/10 (100%)	9/10(90%)	p=0.368
Marks Obtained	35/40 (87.5%)	36/40 (90.0%)	33/40 (82.5%)	p=0.368
Total Score	82/100 (82.0%)	84/100 (84.0%)	82/100 (82.0%)	p=0.368

* Since the scores are equal across all models and there is no variation, Kruskal-Walli’s test cannot be performed. However, we can manually assign a p-value of 1 (indicating no difference).

## DISCUSSION

In recent years, AI tools have transformed the landscape of industries, healthcare systems, academia, and scientific research. AI has made significant strides in revolutionizing medical sciences and healthcare settings.^1^,^12^ AI holds enormous promise to enhance the medical education and healthcare delivery system and improve patient care services. The present study examined the medical knowledge, validity, and reliability of DeepSeek-R1, ChatGPT-4, and Google Gemini 1.5 Pro in medical sciences settings. This is the first study to be added to medical literature and highlights the knowledge of DeepSeek-R1, ChatGPT-4, and Google Gemini 1.5 Pro. The results reveal that all these AI models ChatGPT-4, Google Gemini 1.5 Pro, and DeepSeek R1, obtained similar scores in the medical sciences examination. It supports the validity and reliability of these tools in medical education (Table-II).

There has been significant debate in print, electronic media, the public, the science community, and industrial circles about the Chinese-based AI language model “DeepSeek”. It is less expensive than US-based OpenAI, such as ChatGPT and Google Gemini.^5^ The science community is discussing the knowledge, reliability, and validity level of DeepSeek-R1 and other AI models. In this study, we got all these answers. These AI models, including DeepSeek-R1, ChatGPT-4, and Google Gemini 1.5 Pro, obtained similar scores in medical sciences examinations in diverse disciplines.

The scenario-based MCQs offer a higher cognitive-based evaluation of the applicant’s knowledge and capabilities. Few studies have been published worldwide on ChatGPT’s knowledge assessment, and these researchers reported almost similar performance of ChatGPT. Passby et al. 2023^13^, Duong et al. 2023^14^, Wang et al. 2023^15^, Suchman et al. 2023^16^, Gupta et al. 2023^17^, Jeong et al., 2024^18^, and Nguyen et al. 2025^19^ investigated the knowledge of ChatGPT. It was found that ChatGPT achieved an overall score of 63.1%, 68.2%, 67.6%, 62.4%, 54.96%, 65.4% and 83.8%, respectively. Similarly, the literature highlights the knowledge of Google Bard, its advanced version Gemini, on MCQs examinations. Jeong et al. 2024 (50.0%);^18^ Nguyen et al. 2025 (77.1%);^19^ Mavrych et al. 2025 (53.6%);^20^ and Altamimi et al., 2024 (46%)^21^, these studies investigated the medical knowledge of Google Gemini and found that Gemini’s overall score is slightly lower than the ChatGPT’s. Furthermore, the literature highlights that Google Bard can identify and interpret medical figures, scans, and images and respond to about 56% of the correct image and scan identifications in medicine and allied sciences.^22^

No single study is available on the knowledge assessment of DeepSeek-R1, ChatGPT-4 and Google Gemini 1.5 Pro in medical examinations. In the present study, we found that DeepSeek-R1 performed similarly to ChatGPT-4 and Google Gemini-1.5 Pro and achieved an overall 82% score in the various basic and clinical sciences disciplines in the MCQ-based examination. We believe this is the remarkable success of DeepSeek, an inexpensive and freely available LLM; it can close the gap with the world’s best LLMs, such as Chat GPT-4 and Google Gemini. The AI tools demonstrate promising success in assessing and serve as supporting tools in learning processes. These tools are used in clinical and educational purposes.^23^ The newly launched Chinese-based AI language model, DeepSeek, is an efficient large language model similar to OpenAI’s ChatGPT, garnering significant attention like ChatGPT.^24^

The fact is that AI is gradually making its mark in the health sciences, including diagnosis, assessment, image identification, and medical statistical analysis.^25^ These tools play a significant role in medical education, serving as teaching and research assistants, providing rapid information, developing case scenarios, facilitating language translation, offering decision-making support, and enhancing communication.^25^ These tools have had a significant impact on medical science and medical education; however, they cannot be considered a replacement for human capability and knowledge, as they are still limited in their abilities.

The present study’s findings suggest that DeepSeek-R1, ChatGPT-4, and Google Gemini 1.5 Pro are influential tools for question-answering across various disciplines in the medical sciences. In this study, all three AI models, DeepSeek-R1, ChatGPT-4, and Google Gemini 1.5 Pro, accurately selected the correct option from the multiple-choice preferences. These AI tools ignored the inaccurate answer choices and selected the correct selection. This feature demonstrates the appropriate knowledge levels and the ability to distinguish between similar concepts, identifying misconceptions or gaps in understanding. It shows that DeepSeek-R1, ChatGPT-4, and Google Gemini 1.5 Pro possess distinct medical knowledge, with broader understanding, higher-order thinking, and problem-solving capabilities in the medical sciences. The implications of the findings suggest that DeepSeek, ChatGPT and Gemini are good tools and sources of knowledge; however, the practical application must be carefully regulated.

### Study strengths and limitations:

This is the first novel study to assess and compare the levels of knowledge and understanding of three different AI tools, DeepSeek-R1, ChatGPT-4, and Google Gemini 1.5 Pro in various disciplines of medical sciences. In the future, these AI tools may be highly beneficial in medical education, research, and healthcare settings. The limitation of this study is that we were unable to compare it with other AI tools and their performance in real-world settings, as we routinely perform in classroom settings.

## CONCLUSIONS

The newly launched Chinese-based DeepSeek R1, US-based ChatGPT-4, and Google Gemini- 1.5 Pro obtained similar scores with almost parallel knowledge, validity, and reliability levels in MCQ-based assessments in various medical science disciplines. In the future, these AI models will support medical faculty, physicians, researchers, and scientists across multiple domains of medical science, medical education, and healthcare settings.

## References

[1] Meo SA, Al-Masri AA, Alotaibi M, Meo MZS, Meo MOS (2023). ChatGPT Knowledge Evaluation in Basic and Clinical Medical Sciences:Multiple Choice Question Examination-Based Performance. Healthcare (Basel).

[2] Sun L, Yin C, Xu Q, Zhao W (2023). Artificial intelligence for healthcare and medical education:a systematic review. Am J Transl Res.

[3] Gibney E (2025). China's cheap, open AI model DeepSeek thrills scientists. Nature.

[4] Conroy G, Mallapaty S (2025). How China created AI model DeepSeek and shocked the world. Nature.

[5] Gibney E (2025). Scientists flock to DeepSeek:how they are using the blockbuster AI model. Nature.

[6] Tutor AS, Escudero E, Del Nogal Ávila M, Aranda JF, Torres H, Yague JG (2023). Learning, and assessment strategies to develop specific and transversal competencies for a humanized medical education. Front Physiol.

[7] Palmer EJ, Devitt PG (2007). Assessment of higher order cognitive skills in undergraduate education:modified essay or multiple-choice questions?. BMC Med Educ.

[8] Mingo MA, Chang H, Williams RL (2018). Undergraduate students'preferences for constructed versus multiple-choice assessment of learning. Innov Higher Educ.

[9] Bhayana R, Krishna S, Bleakney RR (2023). Performance of ChatGPT on a radiology board-style examination:insights into current strengths and limitations. Radiology.

[10] Pearson H (2024). Can AI review the scientific literature and determine what it all means?. Nature.

[11] Jones N (2025). OpenAI's 'deep research'tool:Is it helpful for scientists?. Nature.

[12] Alowais SA, Alghamdi SS, Alsuhebany N, Alqahtani T, Alshaya AI, Almohareb SN (2023). Revolutionizing healthcare:the role of artificial intelligence in clinical practice. BMC Med Educ.

[13] Passby L, Jenko N, Wernham A (2024). Performance of ChatGPT on Specialty Certificate Examination in Dermatology multiple-choice questions. Clin Exp Dermatol.

[14] Duong D, Solomon BD (2024). Analysis of large-language model versus human performance for genetics questions. Eur J Hum Genet.

[15] Wang YM, Shen HW, Chen TJ (2023). Performance of ChatGPT on the pharmacist licensing examination in Taiwan. J Chin Med Assoc.

[16] Suchman K, Garg S, Trindade AJ (2023). Chat Generative Pretrained Transformer Fails the Multiple-Choice American College of Gastroenterology Self-Assessment Test. Am J Gastroenterol.

[17] Gupta R, Herzog I, Park JB, Weisberger J, Firouzbakht P, Ocon V (2023). Performance of ChatGPT on the Plastic Surgery Inservice Training Examination. Aesthet Surg J.

[18] Jeong H, Han SS, Yu Y, Kim S, Jeon KJ (2024). How well does large language model-based chatbots perform in oral and maxillofacial radiology?. Dentomaxillofac Radiol.

[19] Nguyen HC, Dang HP, Nguyen TL, Hoang V, Nguyen VA (2025). Accuracy of latest large language models in answering multiple choice questions in dentistry:A comparative study. PLoS One.

[20] Mavrych V, Yaqinuddin A, Bolgova O (2025). Claude, ChatGPT, Copilot, and Gemini Performance versus Students in Different Topics of Neuroscience. Adv Physiol Educ.

[21] Altamimi I, Alhumimidi A, Alshehri S, Alrumayan A, Al-Khlaiwi T, Meo SA (2024). The scientific knowledge of three large language models in cardiology:multiple-choice questions examination-based performance. Ann Med Surg (Lond).

[22] Meo SA, AbuKhalaf AA, Meo MZS, Meo MOS, Ayub R, ElToukhy RA (2024). Role of artificial intelligence (Google bard) in morphological, histopathological, and radiological image identifications:Objective Structured Practical Examination (OSPE) type-based performance. Saudi Med J.

[23] Aksoy I, Arslan MK (2025). Comparison of performance of artificial intelligence tools in answering emergency medicine question pool:ChatGPT 4.0, Google Gemini and Microsoft Copilot. Pak J Med Sci.

[24] Normile D (2025). Chinese firm's large language model makes a splash. Science.

[25] Meo AS, Shaikh N, Meo SA (2024). Assessing the accuracy and efficiency of Chat GPT-4 Omni (GPT-4o) in biomedical statistics:Comparative study with traditional tools. Saudi Med J.

[26] Khan RA, Jawaid M, Khan AR, Sajjad M (2023). ChatGPT - Reshaping medical education and clinical management. Pak J Med Sci.

